# The association between triglyceride glucose-body mass index and all-cause and cardiovascular mortality in diabetes patients: a retrospective study from NHANES database

**DOI:** 10.1038/s41598-024-63886-z

**Published:** 2024-06-16

**Authors:** Shucai Xiao, Qin Zhang, Hai-Yue Yang, Jin-Ying Tong, Ren-Qiang Yang

**Affiliations:** 1 Department of Cardiovascular Medicine, The Second Affiliated Hospital, Jiangxi Medical College, Nanchang University, Nanchang, 330006 Jiangxi China; 2 Department of Metabolism and Endocrinology, The Second Affiliated Hospital, Jiangxi Medical College, Nanchang University, Nanchang, 330006 Jiangxi China; 3 Cardiology Division, Department of Medicine, Queen Mary Hospital, University of Hong Kong, Hong Kong, China; 4 Jiangxi Yingtan People’s Hospital, Yingtan, 335099 Jiangxi China

**Keywords:** Triglyceride glucose-body mass index, Diabetes, Mortality, Cardiovascular disease, NHANES, Cardiology, Endocrinology, Medical research, Risk factors

## Abstract

The triglyceride glucose body mass index (TyG-BMI) is a potential indicator for insulin resistance, but its association with mortality in diabetic patients is unclear. This study investigates the relationship between TyG-BMI and all-cause and cardiovascular mortality in diabetics. The study included 3109 diabetic patients from the National Health and Nutrition Examination Survey (2001–2018). Mortality data were obtained from National Death Index records until 31 December 2019. Multivariate Cox models analyzed the association between TyG-BMI and mortality. Non-linear correlations were assessed using restricted cubic splines, and a two-piecewise Cox model evaluated the relationship on both sides of the inflection point. Over a median 7.25-year follow-up, 795 total and 238 cardiovascular deaths occurred. A U-shaped link was found between initial TyG-BMI and mortality in diabetic patients. Low TyG-BMI (< 279.67 for all-cause, < 270.19 for CVD) reduced death risks (all-cause: HR 0.77, 95% CI 0.69–0.86; CVD: HR 0.64, 95% CI 0.48–0.86). High TyG-BMI (> 279.67 for all-cause, > 270.19 for CVD) increased these risks (all-cause: HR 1.26, 95% CI 1.10–1.44; CVD: HR 1.33, 95% CI 1.06–1.68). In the NHANES study population, a U-shaped association was observed between the baseline TyG-BMI index and all-cause mortality or CVD in diabetic patients.

Diabetes has emerged as a significant global health issue in recent decades^1,2^. According to a recent survey by the International Diabetes Federation (IDF), 536.6 million people worldwide suffered from diabetes in 2021, and it is estimated that the number of diabetic patients worldwide will reach 700 million by 2045^1^. It is reported that diabetes is associated with an increased risk of a variety of complications, including cardiovascular diseases, kidney diseases, and neuropathy^3–5^. In addition, the all-cause mortality and cardiovascular mortality of diabetic patients increased significantly^6^. Therefore, timely identification of more risk factors is crucial to prevent, delay or reduce the progression of diabetes and diabetes related deaths^7^.

Insulin resistance (IR) is a key risk factor for type 2 diabetes mellitus (T2DM), dyslipidemias, obesity, and cardiovascular disease (CVD)^8^. It's linked to CVD progression and cardiovascular outcomes prediction^9^. The triglyceride-glucose (TyG) index, calculated as Ln(fasting triglycerides [mg/dl] × fasting plasma glucose [mg/dl]/2), assesses IR^10^. This index, in contrast to complex methods like the hyperinsulinemic euglycemic clamp (HIEC) and Homeostasis Model of Insulin Resistance (HOMA-IR), is more accessible and cost-effective^11^. Studies demonstrate its close relationship with glucose clamp techniques, endorsing its clinical use as an IR marker^12^. It's noted that TyG is more strongly associated with IR than HOMA-IR^13^ and is linked to CVD^14^ and negative outcomes in ACS, heart failure (HF), and ischemic stroke^15–17^. Recently, some scholars have proposed triglyceride glucose-body mass (TyG-BMI) index, which combines glucose metabolism indicators, lipid metabolism indicators, and anthropometric indicators. After comparing traditional blood lipid parameters, glucose parameters, and obesity related indicators, it was found that TyG-BMI has high diagnostic value in identifying IR^18^. However, there remains debate over the TyG-BMI index's ability, as an IR marker, to forecast outcomes in diabetic patients. Hence, our goal is to investigate the prognostic significance of the TyG-BMI index in assessing the risk of all-cause and CVD mortality among diabetic patients.

## Methods

### Study population and design

**Figure 1:**
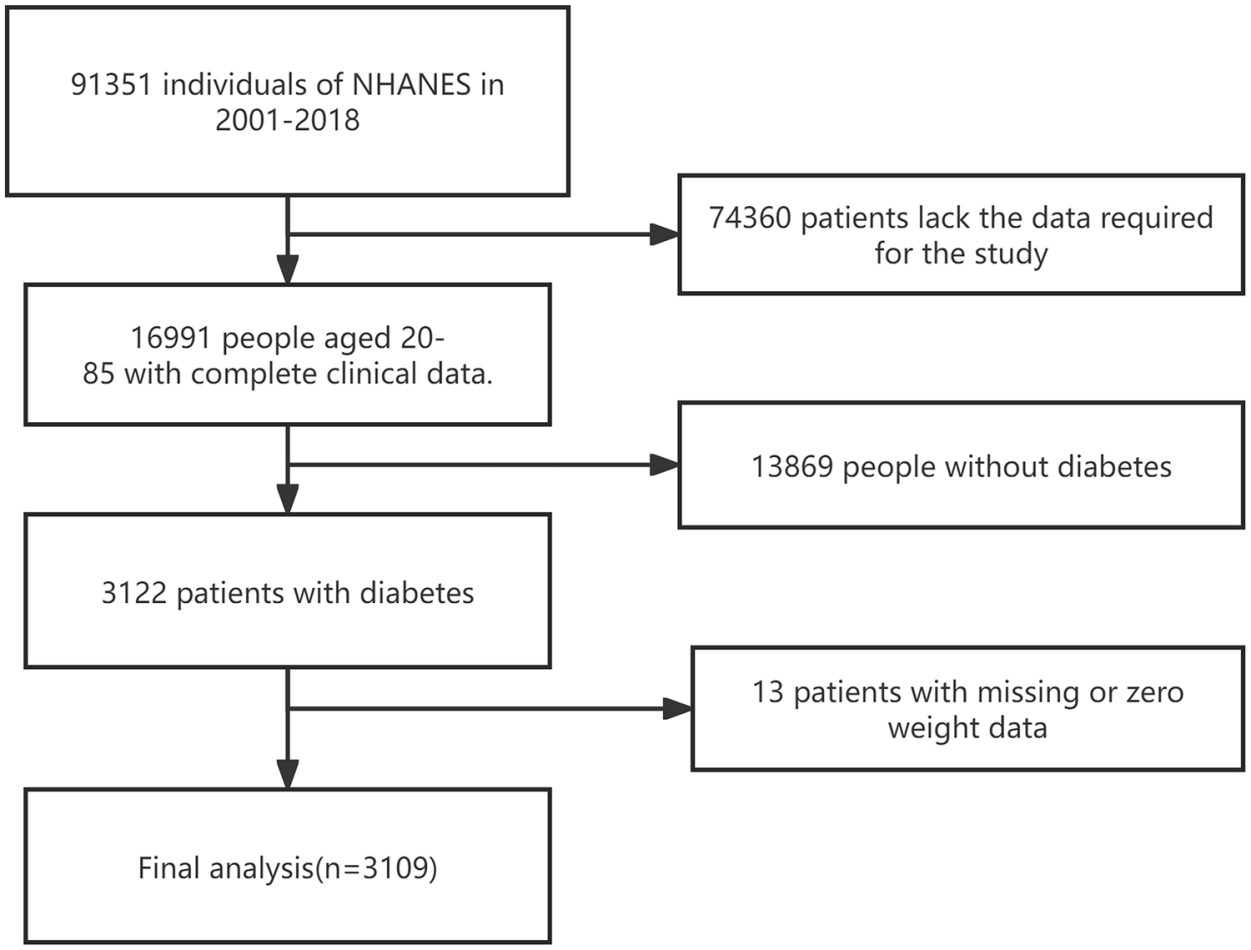
Flow chart of study participants.

The National Health and Nutrition Examination Survey (NHANES) assesses the health and nutrition of U.S. adults and children. Overseen by the Centers for Disease Control and Prevention (CDC), NHANES's protocols are approved by the National Center for Health Statistics (NCHS) Research Ethics Review Board. Participant rights are protected through informed consent. Data for this study, spanning 2001–2018, is from NHANES's website (https://www.cdc.gov/nchs/nhanes/index.html). Diabetes was defined following ADA criteria as a self-reported diagnosis, insulin or hypoglycemic medication use, FBG ≥ 126 mg/dL, or HbA1c ≥ 6.5%^19^. We examined 3122 diabetic adults aged 20–85 years. Excluding 13 without baseline TyG-BMI index data, 3109 were included in our analysis (Fig. 1).

### Assessment of covariates

We obtained patient histories at baseline. Demographics including age, sex, race/ethnicity, education level, family income, smoking status, comorbidities (diabetes, CVD, hypertension, chronic obstructive pulmonary disease). The diagnosis of CVD was established by self-reported physician diagnoses obtained during an individual interview using a standardized medical condition questionnaire. The participants were asked, “Has a doctor or other health expert ever informed you that you have CHF/CHD/angina pectoris/MI/stroke?” A person was regarded as having CVD if he or she replied “yes” to any of the above questions. Body mass index (BMI) was calculated using weight (kg) divided by height (m^2). Participants' race/ethnicity included White, Black, Mexican, or Other, and education levels were divided into less than high school, high school/equivalent, or college and above. Income and poverty were classified into three categories: 0–1.0, 1.0–3.0, or > 3.0. Smoking status was identified as never, former, or current smoker. Drinking was categorized into heavy (≥ 3 drinks daily for women, ≥ 4 for men, or binge drinking [≥ 4 drinks for women, ≥ 5 for men] on 5 + days/month), moderate (≥ 2 drinks daily for women, ≥ 3 for men, or binge drinking ≥ 2 days/month), mild (not meeting the above), nondrinker, or history of daily binge drinking.

### Assessment of TyG-BMI index

The TyG-BMI index was derived by the formula: ln[TG (mg/dL) × FPG (mg/dL)/2] × BMI (kg/m^2), where TG stands for triglycerides and FBG for fasting blood glucose. Triglycerides were quantified using enzymatic assays conducted on a Roche Modular P analyzer, while FBG levels were determined through a hexokinase-mediated enzymatic method on a Roche/Hitachi Cobas C 501 analyzer. To facilitate comparative analysis, the study population was stratified into quartiles (Q1 to Q4) based on the TyG-BMI index distribution, with the lowest quartile (Q1) serving as the reference cohort.

### Ascertainment of mortality

To determine mortality in the follow-up group, we used the NHANES public-use linked mortality file as of December 31, 2019. This file is connected to the National Death Index (NDI) through a probability matching algorithm by NCHS. We defined CVD mortality using the International Statistical Classification of Diseases and Related Health Problems, Tenth Revision codes I00 to I09, I11, I13, I20 to I51, and I60 to I69^20^.

### Statistical analysis

Statistical analyses were performed using R software (version 4.2.1; https://www.r-project.org). To accommodate NHANES's complex sampling method, sample weights, clustering, and stratification were integrated into all analyses^21^. Participants were segmented into quartiles (Q1-Q4) according to their TyG-BMI index. We presented continuous variables as means and SDs and categorical variables as frequencies and percentages. Differences in baseline characteristics by TyG-BMI quartiles were assessed using one-way ANOVA for continuous and Pearson chi-square test for categorical variables.

All-cause and CVD mortality rates were computed for each TyG-BMI quartile during the follow-up. To assess the predictive power of TyG-BMI, we employed various Cox proportional hazards regression models. These comprised an unadjusted model (Model 1), a model adjusted for age, race, and gender (Model 2), and a fully adjusted model (Model 3) that also included variables such as tobacco and alcohol use, education level, hypertension, COPD, CVD, eGFR, HDL, FINS and family income-poverty ratio. The relationship between the TyG-BMI index and mortality was analyzed using multivariable adjusted Cox models, employing restricted cubic splines and penalized spline methods for smooth curve fitting. Non-linear relationships prompted threshold estimation through likelihood maximization, followed by a two-piecewise Cox model on either side of the inflection point. Kaplan–Meier plots and log-rank tests compared survival rates among different TyG-BMI index categories. We also performed subgroup analyses, taking into account variables like gender, age (either < 60 or ≥ 60 years), race (White, Black, Mexican, or Other), COPD, CVD and hypertension. A p-value below 0.05 was considered statistically significant.

### Ethical approval

The protocol for NHANES received approval from the National Center for Health Statistics and Ethics Review Board, with all participants giving written informed consent.

## Results

### Baseline characteristics of study participants

Table 1 outlines the initial characteristics of the study group (n = 3109), divided by TyG-BMI index quartiles. The average age was 58.93 years, and males constituted 51.19%. Baseline lab results are detailed in Table 2. Participants with higher TyG-BMI indices were generally younger, had higher obesity levels, and showed greater prevalence of hypertension compared to the lowest quartile.

**Table 1:** Baseline characteristics according to the TyG-BMI index quartiles.

Characteristics	Quartiles of TyG-BMI index					*P* value
	Overall	Q1 (119.61–241.61)	Q2 (241.61–282.74)	Q3 (282.74–334.26)	Q4 (334.26–710.26)	
N (%)	3109	778 (25.02)	777 (24.99)	776 (24.96)	778 (25.02)	
Age, years, mean (SD)	58.93 (0.34)	63.48 (0.71)	62.00 (0.61)	58.24 (0.62)	53.64 (0.57)	< 0.0001
Gender, n (%)						0.07
Female	1468 (48.81)	323 (44.55)	334 (47.13)	383 (48.33)	428 (53.88)	
Male	1641 (51.19)	455 (55.45)	443 (52.87)	393 (51.67)	350 (46.12)	
BMI, kg/m^2^, n (%)	32.73 (0.19)	24.48 (0.11)	29.16 (0.09)	33.12 (0.15)	41.61 (0.27)	< 0.0001
Race, n (%)						< 0.0001
Black	692 (13.32)	150 (11.94)	180 (15.04)	167 (11.64)	195 (14.62)	
Mexican	598 (9.00)	105 (5.90)	154 (8.65)	191 (11.82)	148 (9.16)	
White	549 (12.18)	189 (18.24)	145 (12.42)	119 (10.63)	96 (8.62)	
Other	1270 (65.50)	334 (63.92)	298 (63.88)	299 (65.91)	339 (67.60)	
Education, n (%)						0.31
High school grad or equivalent	763 (27.51)	201 (28.27)	189 (25.29)	170 (25.63)	203 (30.28)	
Less than high school	1030 (22.36)	254 (23.68)	265 (23.71)	286 (23.79)	225 (19.02)	
Some college or above	1316 (50.13)	323 (48.04)	323 (51.00)	320 (50.58)	350 (50.70)	
Alcohol, n (%)						0.52
Former	805 (22.81)	193 (21.33)	200 (20.65)	216 (24.22)	196 (24.35)	
Heavy	428 (14.01)	92 (12.77)	101 (14.03)	108 (13.25)	127 (15.65)	
Mild	1002 (34.82)	267 (36.78)	265 (37.46)	244 (34.80)	226 (31.28)	
Moderate	323 (12.46)	75 (10.45)	80 (13.14)	80 (13.90)	88 (12.24)	
Never	551 (15.90)	151 (18.67)	131 (14.72)	128 (13.82)	141 (16.49)	
Smoking status, n (%)						0.19
Former	1030 (33.41)	225 (28.93)	276 (36.71)	284 (36.46)	245 (31.70)	
Never	1558 (49.27)	405 (52.02)	374 (45.65)	371 (47.33)	408 (51.60)	
Now	521 (17.32)	148 (19.05)	127 (17.64)	121 (16.21)	125 (16.71)	
Family poverty income ratio, mean (SD)	2.76 (0.05)	2.68 (0.08)	2.85 (0.08)	2.88 (0.09)	2.67 (0.08)	0.19
Hypertension, n (%)						< 0.001
No	891 (30.35)	271 (37.42)	224 (33.41)	207 (27.11)	189 (25.35)	
Yes	2218 (69.65)	507 (62.58)	553 (66.59)	569 (72.89)	589 (74.65)	
COPD, n (%)						0.07
No	2901 (93.20)	716 (90.62)	734 (94.43)	718 (92.96)	733 (94.52)	
Yes	208 (6.80)	62 (9.38)	43 (5.57)	58 (7.04)	45 (5.48)	
eGFR	84.77 (0.53)	80.20 (1.02)	83.01 (1.14)	84.84 (1.15)	89.66 (0.99)	< 0.0001
CVD						0.98
No	2373 (77.72)	598 (77.55)	590 (78.40)	592 (77.87)	593 (77.19)	
Yes	736 (22.28)	180 (22.45)	187 (21.60)	184 (22.13)	185 (22.81)	

Data are presented as mean (SD) or n (%);

*COPD* chronic obstructive pulmonary disease, *eGFR* estimated glomerular filtration rate, *CVD* cardiovascular disease, *SD* Standard deviation.

**Table 2:** Baseline levels of laboratory characteristics according to the TyG-BMI index quartiles.

	Quartiles of TyG-BMI index				
	Q1 (119.61–241.61)	Q2 (241.61–282.74)	Q3 (282.74–334.26)	Q4 (334.26–710.26)	*P* value
LDL-cholesterol, mmol/L, mean (SD)	2.72 (0.04)	2.80 (0.04)	2.85 (0.05)	2.81 (0.05)	0.24
HDL-cholesterol, mmol/L, mean (SD)	1.50 (0.03)	1.30 (0.02)	1.19 (0.01)	1.14 (0.01)	< 0.0001
TC, mmol/L, mean (SD)	4.78 (0.05)	4.84 (0.05)	4.94 (0.06)	5.02 (0.06)	0.01
TG, mmol/L, mean (SD)	1.23 (0.03)	1.64 (0.04)	2.07 (0.09)	2.57 (0.13)	< 0.0001
Scr, umol/L, mean (SD)	93.41 (3.58)	85.10 (2.36)	82.20 (1.24)	81.39 (1.69)	0.02
LDH, IU/L, mean (SD)	135.49 (1.53)	133.93 (1.57)	133.36 (1.54)	139.39 (1.72)	0.06
Uric acid, umol/L, mean (SD)	322.31 (3.47)	342.37 (4.42)	358.86 (4.44)	364.79 (4.52)	< 0.0001
Albumin, g/L, mean (SD)	42.63 (0.18)	42.03 (0.14)	41.51 (0.16)	40.11 (0.16)	< 0.0001
FINS, pmol/L, mean (SD)	60.55 (2.70)	96.57 (7.02)	123.21 (6.29)	179.48 (8.14)	< 0.0001
FPG, mmol/L, mean (SD)	7.33 (0.14)	7.77 (0.12)	8.31 (0.14)	9.16 (0.16)	< 0.0001
Serum potassium, mmol/L, mean (SD)	4.12 (0.02)	4.09 (0.02)	4.09 (0.02)	4.11 (0.02)	0.64
Serum iron, umol/L, mean (SD)	16.21 (0.29)	16.21 (0.26)	15.23 (0.28)	13.94 (0.25)	< 0.0001
Serum sodium, mmol/L, mean (SD)	139.33 (0.14)	139.11 (0.12)	138.99 (0.16)	138.49 (0.16)	< 0.001
BUN, mmol/L, mean (SD)	5.89 (0.10)	5.60 (0.13)	5.57 (0.09)	5.45 (0.12)	0.03
AST (IU/L)	25.74 (0.59)	25.52 (0.61)	27.39 (0.90)	27.20 (0.61)	0.16
ALT (IU/L)	23.50 (0.57)	26.90 (0.86)	30.20 (0.89)	30.69 (1.04)	< 0.0001
TBil, umol/L, mean (SD)	12.94 (0.24)	12.22 (0.24)	12.17 (0.28)	11.07 (0.24)	< 0.0001
GGT (IU/L)	32.95 (1.77)	39.01 (3.17)	37.66 (1.73)	46.69 (4.81)	0.02

Data are presented as mean (SD) or n (%);

### Relationships of TyG-BMI index with mortality

**Figure 2:**
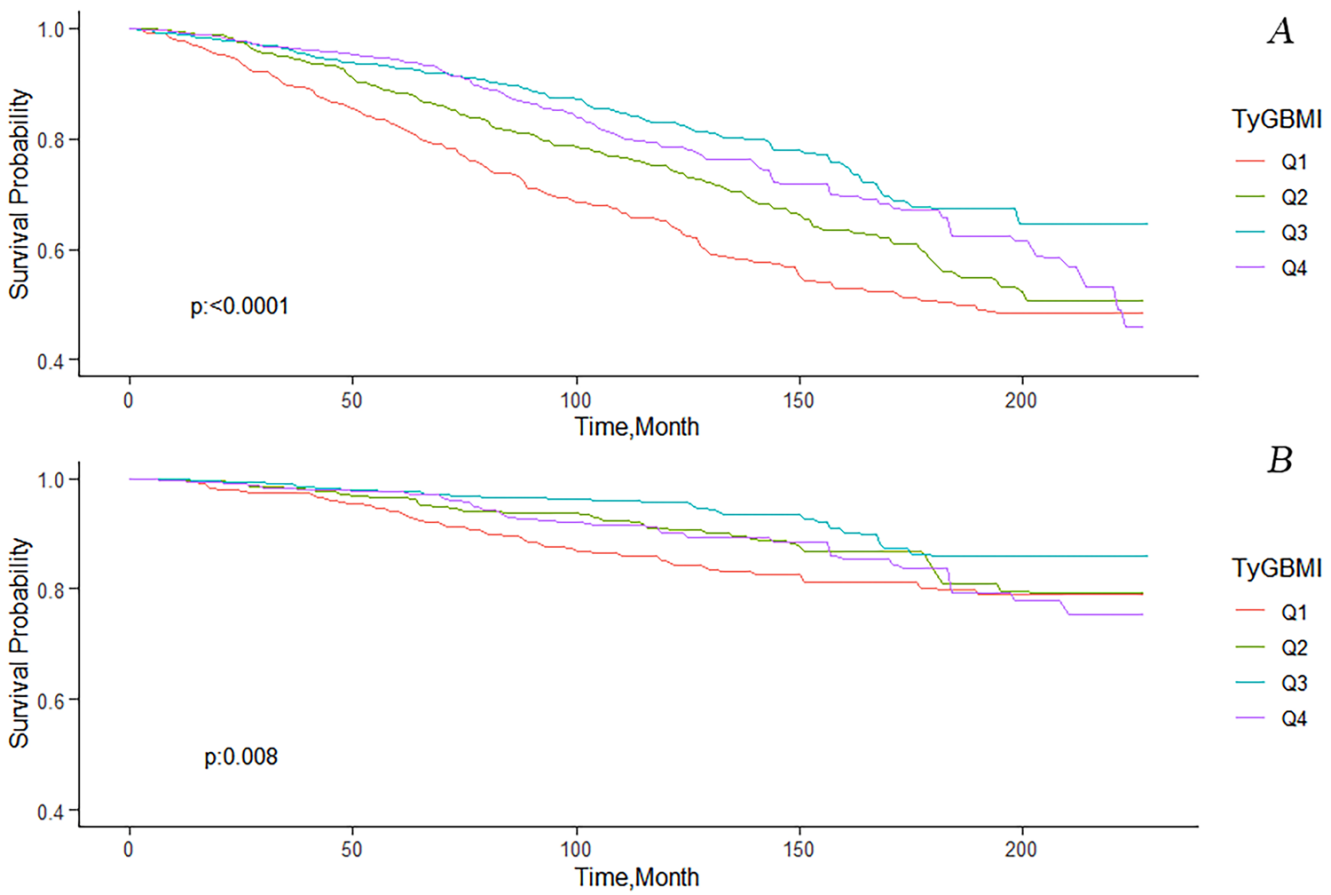
Kaplan–Meier curves of the survival rate and the number (%) of at-risk diabetes patients. TyG-BMI Index Quartile 1 was used as the Reference group. (**A**) All-cause mortality, (**B**) cardiovascular mortality.

Kaplan Meier curves indicated lower all-cause and CVD mortality in patients with elevated TyG-BMI compared to lower levels (*p* < 0.0001) (Fig. 2). There were 795 all-cause and 238 CVD deaths during follow-up (Table 3). We used three Cox models to assess the link between TyG-BMI levels and mortality risk. In Model 3, adjusted for various factors, multivariate-adjusted HRs and 95% CIs for all-cause mortality across TyG-BMI quartiles (119.61–241.61, 241.61–282.74, 282.74–334.26, 334.26–710.26) were 1.00 (reference), 0.72 (0.59,0.88), 0.56 (0.45,0.70), and 0.86 (0.68,1.09) (P = 0.046); for CVD mortality, they were 1.00 (reference), 0.65 (0.44,0.97), 0.56 (0.33,0.92), and 1.15 (0.78,1.69) (*P* = 0.646).

**Table 3:** HRs (95% CIs) for mortality according to the TyG-BMI index quartiles.

	Quartiles of TyG-BMI index						
	Q1 (119.61–241.61)	Q2 (241.61–282.74)		Q3 (282.74–334.26)	Q4 (334.26–710.26)		*P* trend
All-cause mortality							
Number of deaths	259	213	164		159		
Model 1	1	0.70 (0.55, 0.88) 0.002	0.45 (0.35, 0.57) < 0.0001		0.53 (0.43, 0.65) < 0.0001	< 0.0001	
HR (95% CI) *P*-value							
Model 2	1	0.74 (0.60, 0.90) 0.003	0.62 (0.51, 0.77) < 0.0001		0.97 (0.80, 1.18) 0.78	0.276	
HR (95% CI) *P*-value							
Model 3	1	0.72 (0.59, 0.88) 0.001	0.56 (0.45, 0.70) < 0.0001		0.86 (0.68, 1.09) 0.2	0.046	
HR (95% CI) *P*-value							
CVD mortality							
Number of deaths (%)	71	62	50		55		
Model 1	1	0.66 (0.46, 0.96) 0.03	0.42 (0.26, 0.69) < 0.001		0.70 (0.49, 0.98) 0.04	< 0.038	
HR (95% CI) *P*-value							
Model 2	1	0.70 (0.49, 0.98) 0.04	0.60 (0.38, 0.95) 0.03		1.29 (0.93, 1.81) 0.13	0.315	
HR (95% CI) *P*-value							
Model 3	1	0.65 (0.44, 0.97) 0.03	0.56 (0.33, 0.92) 0.02		1.15 (0.78, 1.69) 0.48	0.646	
HR (95% CI) *P*-value							

Model 1: Non-adjusted.

Model 2: Adjusted for age, race and gender.

Model 3: Adjusted for age, gender, race, tobacco use, alcohol use, education, hypertension, COPD, CVD, eGFR, family income-poverty ratio, HDL, FINS. HR: Hazard ratio; CI: Confidence interval. COPD: chronic obstructive pulmonary disease, eGFR: estimated glomerular filtration rate, CVD: cardiovascular disease, HDL:High density lipoprotein, FINS:Fasting insulin.

### The detection of nonlinear relationships

**Figure 3:**
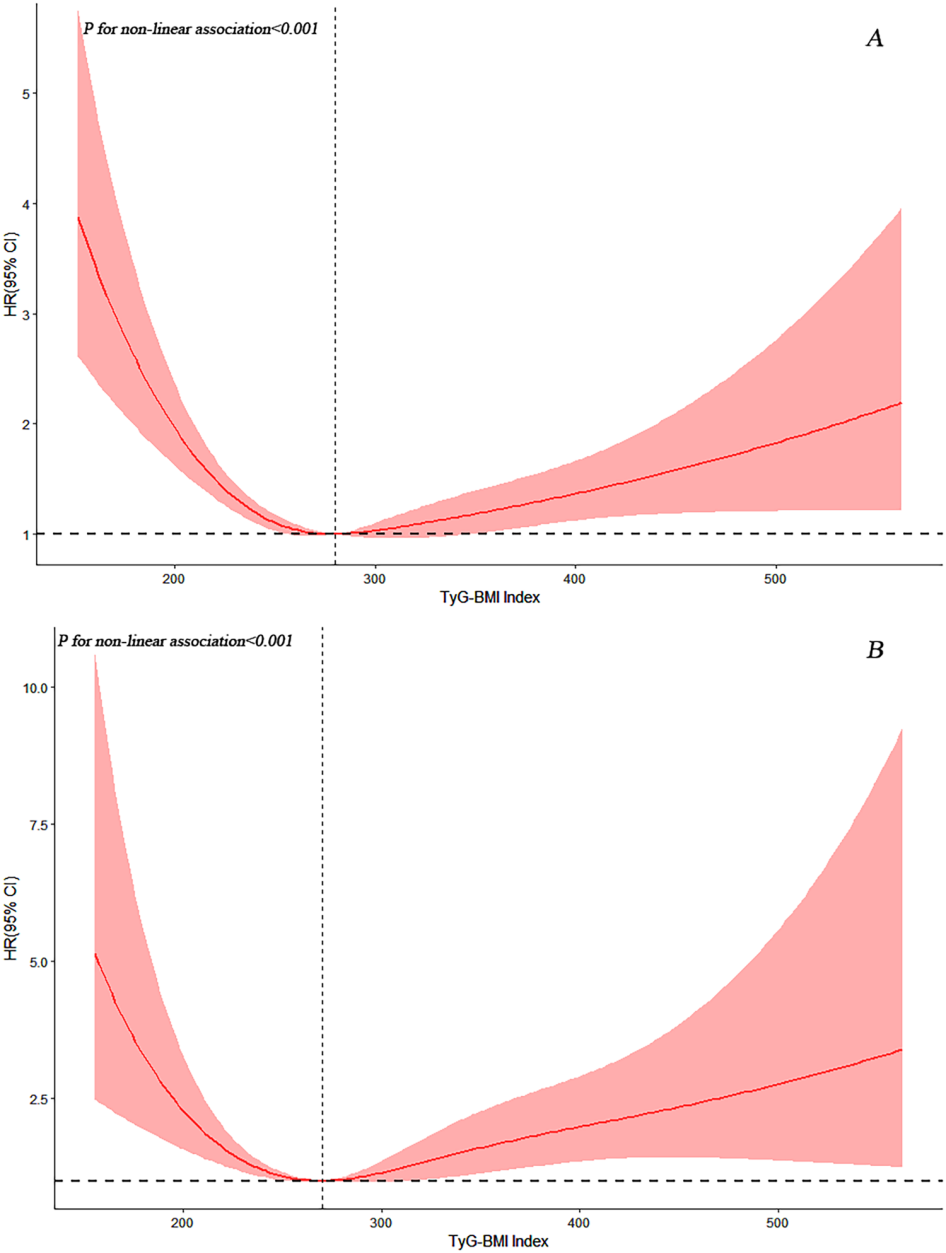
Association between TyG-BMI index and all-cause (**A**) and CVD mortality (**B**) in patients with diabetes. Each hazard ratio was computed with a TyG-BMI index level of A 279.67 and B 270.19 as the reference. Adjusted for age, gender, race, tobacco use, alcohol use, education, hypertension, COPD, CVD, eGFR, family income-poverty ratio, HDL, FINS. The solid line and red area represent the estimated values and their corresponding 95% CIs, respectively (TyG-BMI Index: Triglyceride glucose-body mass index; CVD: cardiovascular disease).

Prior analysis identified a non-linear relationship between initial TyG-BMI index and mortality risks from all causes and CVD, using of Cox proportional hazards regression with restricted cubic splines for smooth curves. Adjusted results revealed U-shaped patterns between TyG-BMI index and both all-cause (Fig. 3A) and CVD mortality (Fig. 3B). Using both standard and two-piecewise Cox models, we located inflection points at 279.67 for all-cause and 270.19 for CVD mortality (log-likelihood ratio *P* values < 0.001) (Table 4). Before reaching the above threshold, the baseline TyG-BMI index had the lowest risk (Table 4 and Fig. 3). For each standard deviation increase in the TyG-BMI index, the corresponding all-cause mortality and CVD mortality rates decreased by 23% (HR 0.77, 95% CI 0.69–0.86) and 36% (HR 0.64, 95% CI 0.48–0.86), respectively. However, after reaching the above threshold, each standard deviation increase in the TyG-BMI index increased the risk of all-cause mortality (HR 1.26, 95% CI 1.10–1.44) and CVD mortality (HR 1.33, 95% CI = 1.06–1.68).

**Table 4:** Threshold effect analysis of TyG index on all-cause and CVD mortality in CVD patients with diabetes or pre-diabetes.

	Adjusted HR (95% CI), *P*-value
All-cause mortality	
Total, per 1 SD	0.92 (0.82,1.04) 0.17
Fitting by two-piecewise Cox proportional risk model	
Inflection point	279.67
TyG-BMI index < 279.67, per 1 SD	0.77 (0.69,0.86) < 0.0001
TyG-BMI index ≥ 279.67, per 1 SD	1.26 (1.10,1.44) < 0.001
*P* for Log-likelihood ratio	< 0.001
CVD mortality	
Total, per 1 SD	1.06 (0.85,1.33) 0.60
Fitting by two-piecewise Cox proportional risk model	
Inflection point	270.19
TyG-BMI index < 270.19, per 1 SD	0.64 (0.48,0.86) 0.003
TyG-BMI index ≥ 270.19, per 1 SD	1.33 (1.06,1.68) 0.01
*P* for Log-likelihood ratio	< 0.001

Cox proportional hazards models were used to estimate HR and 95% CI. Adjusted for age, gender, race, tobacco use, alcohol use, education, hypertension, COPD, CVD, eGFR, family income-poverty ratio, HDL, FINS. HR: Hazard ratio; CI: Confidence interval.

### Subgroup analyses

The benefit of a higher TyG-BMI index (≥ 279.67) compared to a lower one (< 279.67) on all-cause mortality in diabetic patients was uniform across subgroups like age, gender, CVD history, and hypertension (Table 5). There was no significant interaction between TyG-BMI index and these variables. However, a stronger inverse relationship was noted between TyG-BMI index and all-cause mortality in diabetic patients under 60. Similarly, a higher TyG-BMI index (≥ 270.19) showed consistent benefits over a lower index (< 270.19) in CVD mortality across subgroups stratified by age, race, COPD, and hypertension history (Table 6). No significant interaction was found between TyG-BMI index and these groups. However, among diabetic women, a more distinct inverse correlation was observed between TyG-BMI index and all-cause mortality.

**Table 5:** Stratified analyses of the associations between TyG-BMI and All mortality.

TyG-BMI index	All-cause mortality				p interaction
	HR(95% CI) *P*-value				
	< 279.67	>=279.67			
Overall	1	0.78(0.66,0.94) 0.01			
Gender					0.015
Female	1	0.59(0.44,0.78) < 0.001			
Male	1	0.98(0.77,1.25) 0.86			
Age, years					0.048
< 60	1	0.96(0.63,1.48) 0.86			
≥ 60	1	0.81(0.67,0.99) 0.04			
Race					0.239
Black	1	0.78(0.56,1.11) 0.16			
Mexican	1	1.06(0.65,1.72) 0.81			
Other	1	1.39(0.63, 3.07) 0.42			
White	1	0.75(0.61,0.92) 0.01			
COPD					0.485
Yes	1	1.67(0.93,2.97) 0.08			
No	1	0.73(0.60,0.87) < 0.001			
Hypertension					0.641
Yes	1	0.76(0.62,0.94) 0.01			
No	1	0.74(0.45,1.23) 0.25			
CVD					0.671
Yes	1	0.71(0.52,0.97) 0.03			
No	1	0.82(0.64,1.04) 0.01

**Table 6:** Stratified analyses of the associations between TyG-BMI and CVD mortality.

TyG-BMI index	CVD mortality		*p* interaction
	HR(95% CI) *P*-value		
	< 270.19	≥ 270.19	
Overall	1	1.01 (0.76,1.35) 0.94	
Gender			0.007
Female	1	0.58 (0.36,0.92) 0.02	
Male	1	1.49 (1.01,2.19) 0.05	
Age, years			0.316
< 60	1	1.56 (0.70, 3.44) 0.27	
≥ 60	1	1.06 (0.75,1.48) 0.75	
Race			0.917
Black	1	1.07 (0.59,1.94) 0.82	
Mexican	1	1.75 (0.48, 6.41) 0.4	
Other	1	1.40 (0.28, 7.12) 0.68	
White	1	1.07 (0.74,1.57) 0.71	
COPD			0.734
Yes	1	8.23 (0.86, 79.01) 0.07	
No	1	0.95 (0.69,1.31) 0.75	
Hypertension			0.785
Yes	1	0.96 (0.68,1.37) 0.84	
No	1	1.25 (0.58, 2.71) 0.57	
CVD			0.97
Yes	1	0.83 (0.45,1.52) 0.54	
No	1	1.16 (0.74, 1.81) 0.52	

## Discussion

In this retrospective cohort study, we found that a U-shaped association between TyG-BMI index and mortalities among diabetic patients was also explored with a turning point at 279.67 for all-cause mortality and 270.19 for CVD mortality. Subgroup analyses of demographics and comorbidities were consistent with these core findings.

Although HOMA-IR is a classic marker of IR^22^, its costliness and complexity have prevented its wide usage. TyG-BMI index is a new indicator proposed in recent years that reflects IR. It combines glucose and lipid metabolism indicators with anthropometric indicators, and has a strong correlation with HOMA-IR, allowing it to make early predictions of IR^18^. The TyG-BMI index, unlike HOMA-IR, doesn't require insulin measurement and is suitable for all patients on insulin therapy. Recent research has linked the TyG-BMI index with heart failure, NAFLD, and hyperuricemia^23–25^. Compared to other IR parameters, a NNHANES study with 9884 participants validated TyG-BMI as a feasible IR evaluation tool^18^. Additionally, an investigation into metabolic indicators and atherosclerotic cardiovascular disease (ASCVD) risk highlighted a notable association between TyG-BMI index and ASCVD risk, particularly in females^26^. Furthermore, Du showed that TyG-BMI index was linearly correlated with ischemic stroke without threshold or saturation effects in the general population^27^. These results, therefore, indicate the potential value of TyG-BMI index as a predictive indicator for metabolic diseases and for risk stratification in patients with diabetes.

Although our study demonstrated a link between TyG-BMI index and mortality in patients with diabetes, the underlying mechanism is still unclear. IR, a key feature of T2DM, is known as a CVD risk factor^28^. It contributes to CVD development in both the general and diabetic populations and is associated with poorer cardiovascular outcomes in CVD patients^29^. Studies indicate that patients with IR are at a heightened risk for metabolic disorders, which are associated with adverse cardiovascular disease outcomes^30^. Persistent hyperglycemia and abnormal blood lipids caused by IR may lead to oxidative stress and inflammatory reactions, thereby damaging endothelial cell function^31^. Continuous IR can increase hypertension and lead to vascular and renal damage^32^. In addition, the levels of free fatty acids (FFA) in plasma can increase with the increase of TG levels, thereby promoting the development of obesity-related IR and CVD^33^. These pathologies collectively may lead to the onset and advancement of coronary heart disease and worsen prognosis.

Our study adds unique insights to the evolving research on the TyG-BMI index, revealing its intricate link with all-cause and CVD mortality risks. We observed that lower baseline TyG-BMI levels (< 279.67 for all-cause, < 270.19 for CVD mortality) significantly influence its relationship with mortality risks. Notably, for those with baseline TyG-BMI below these thresholds, every unit increase in the index is associated with a 24% decrease in all-cause mortality risk and a 36% decrease in CVD mortality risk, post-adjustment for confounders. For example, it is widely recognized that low blood sugar elevates counter-regulatory hormones such as adrenaline, which can cause vasoconstriction and platelet aggregation, leading to an increase in cardiovascular and cerebrovascular stroke events^34^. Similarly, studies on patients with heart failure suggest that low TG levels have been identified as a predictive factor for their cardiac death^35^. In addition, research shows that BMI is associated with mortality in a U-shaped curve regardless of the severity of diabetes^36^. Hence, maintaining a balanced TyG-BMI index is crucial, as both excessively high and low levels could lead to adverse health outcomes.

In subgroup analysis, the impact of TyG-BMI index on all-cause mortality was more obvious in female younger than 60 years old. A plausible reason for this observation is that older men often have multiple illnesses, making a single indicator insufficient to fully assess their effects. Another key discovery from our study is the more pronounced link between the TyG-BMI index and CVD mortality risk in women compared to men. This could be due to estrogen's role in premenopausal cardiovascular protection, which improves insulin sensitivity. A cohort study reported that elevated fasting serum insulin levels and HOMA-IR are linked with hypertension in women, but this association is not seen in men^37^. This study has certain limitations. Due to its single center and observational nature, this study cannot determine causal relationships. Despite adjusting for multiple factors and conducting subgroup analyses, unaccounted confounding factors might influence the prognosis. Furthermore, our analysis concentrated solely on the baseline TyG-BMI index's prognostic value, leaving the effects of TyG-BMI index variations during follow-up on mortality prediction undetermined. Nevertheless, this does not negate the observed association between TyG-BMI index and increased/decreased risk of mortality among patients with diabetes. Further investigations are warranted to confirm such correlation.

## Conclusion

Our study's findings suggest that the TyG-BMI index is an effective predictor of all-cause and CVD mortality risks in diabetic patients, highlighting a non-linear relationship between the TyG-BMI index and mortality. Consequently, utilizing the TyG-BMI index for risk assessment and prognosis prediction in this patient group could be advantageous. Further research is needed to determine if interventions focused on the TyG-BMI index can enhance clinical outcomes in these patients.

## Data Availability

Upon a reasonable request, the datasets utilized and assessed in this study can be acquired from the corresponding author.

## Abbreviations

BMI: Body mass index
CDC: The Centers for Disease Control and Prevention
CHF: Congestive heart failure
CI: Confidence interval
CVD: Cardiovascular disease
DM: Diabetes mellitus
eGFR: Estimated glomerular filtration rate
FINS: Fasting insulin
FFA: Free fatty acid
FPG: Fasting plasma glucose
HDL: High density lipoprotein
HF: Heart failure
HR: Hazard ratio
IR: Insulin resistance
MI: Myocardial infarction
NCHS: National Center for Health Statistics
NDI: National Death Index
NHANES: National Health and Nutrition Examination Survey
SD: Standard deviation
TyG: Triglyceride glucose index
TyG-BMI: Triglyceride glucose body mass index
